# Preparation of Flexible Liquid Crystal Films with Broadband Reflection Based on PD&SLC

**DOI:** 10.3390/ma15248896

**Published:** 2022-12-13

**Authors:** Xuetao Zhang, Rui Han, Hui Li, Xiaohui Zhao, Hui Cao, Yinjie Chen, Zhou Yang, Dong Wang, Wanli He

**Affiliations:** 1 State Key Laboratory for Advanced Metals and Materials, School of Materials Science and Engineering, University of Science and Technology Beijing, Beijing 100083, China; 2 Beijing Engineering Research Center of Printed Electronics, Beijing Institute of Graphic Communication, Beijing 102600, China

**Keywords:** PD&SLC, broadband, flexible film, cholesteric liquid crystal

## Abstract

A simple and efficient method for the preparation of a film with flexible characteristic and selective reflection of near-infrared light is proposed. Based on the coexistence system (PD&SLC) of polymer dispersed liquid crystals (PDLC) and polymer stabilized liquid crystals (PSLC), it combines the flexibility of PDLC with the selectively reflection of PSLC. Innovative use of step-by-step light curing to achieve microstructural differences in the three-dimensional orientation of the material is proposed. That is, the difference between PDLC and PSLC in the planar orientation, as well as the gradient distribution of cholesteric phase liquid crystal pitch in the cell thickness direction, is observed. While realizing the flexibility of the material, the function of broadening the reflection bandwidth is fulfilled. This method of preparing liquid crystal films is expected to have great potential for applications, such as flexible smart windows, infrared light shielding, and sensors.

## 1. Introduction

The coexistence system (PD&SLC) of polymer dispersed liquid crystals (PDLC) and polymer stabilized liquid crystals (PSLC) with new and unique structures and properties can be obtained by artificially changing the microstructure of polymeric liquid crystals [1,2]. PDLCs are liquid crystal/polymer composites in which liquid crystal molecules are encapsulated in a polymer matrix in the form of microdroplets. PDLC films are usually prepared by using the polymeric phase separation method, where small molecules of liquid crystals are homogeneously mixed with non-liquid crystal polymerizable monomers in a certain ratio. The non-liquid crystal polymerizable monomers are then triggered to cross-link by UV irradiation or heating. The increasing molecular chains of polymeric monomers enable the small molecule liquid crystal materials to become less and less soluble in the polymer, resulting in precipitation, incorporation, and growth as liquid crystal microdroplets. When the non-liquid crystal polymerizable monomer is fully cured, the liquid crystal droplets are dispersed in the polymer network as a discontinuous phase [3,4]. The high polymer content of the PDLC system provides excellent bond strength between the two substrates, allowing PDLC to be processed flexibly by using the roll-to-roll method [5].

PSLC and PDLC are both liquid crystal/polymer composites. Unlike the PDLC, the polymeric monomers used in the PSLC are liquid crystal polymerizable monomers. During its curing process, the liquid crystal and liquid crystal polymerizable monomers are pre-oriented, so the liquid crystal polymerizable monomers form a polymeric network inside the liquid crystal, which can stabilize the orientation of the liquid crystal molecules and distinguish it from the porous structure of the PDLC [6]. In addition, where the cholesteric phase liquid crystals are known as chiral nematic liquid crystals (N*-LCs), they can be formed by adding chiral compounds to the nematic liquid crystals [7,8,9,10]. The reflection bandwidth ∆λ depends on the birefringence ∆n and the pitch *P* at normal incidence, and *P* is controlled by the chiral dopant [11]. Broadband reflective liquid crystal films have a wide range of applications and great commercial value [12,13,14,15,16,17,18,19,20,21]. At present, the most attention is paid to energy-saving and environmental protection building glass or paint. This includes smart glass. The near-infrared light energy in the wavelength range of 800–2000 nm accounts for more than 90% of the entire solar infrared radiation energy [22,23,24]. The use of smart glass windows based on PSLC to absorb infrared light can reflect part of the infrared light, which can reduce the increase in indoor heat and reduce the energy consumption of the cooling system [25,26]. Although many methods for broadening the reflective broadband (Δλ) are already available in practical research, as research into flexible materials continues, previous methods can only meet the requirements of rigid devices, and research into flexible films capable of selective reflection is extremely lacking

Yang et al. [27,28] have worked to prepare flexible dimmable liquid crystal films with electrically responsive properties that can be produced on a large scale. A coexisting system combining the good mechanical and processing properties of PDLC with the fast responsive properties of the liquid crystal molecules in PSLC has been proposed.

In summary, this study combines the different advantages of PDLC and PSLC. The PDLC can easily produce large-area flexible films, and the N*-LCs of the PSLC can selectively achieve the reflective broadband [29]. Innovative use of step-by-step light curing to achieve microstructural differences in the three-dimensional orientation of the material is used. First, the liquid crystal film is UV (254 nm), and it is cured under a photo-mask template, which results in a grid-like PDLC polymer wall that effectively prevents the mobility of the liquid crystal during the flexure of the material. It not only increases the stability of liquid crystal molecules and maintains the selective reflection of PSLC, but it also plays a role in the film to resist compression and increase the bond strength between the film and the substrate. The second step removes the mask and performs UV (365 nm) curing of the free radical. Both curing steps result in the migration of chiral compounds due to the mixing of two UV absorbing dyes in appropriate proportions. The gradient distribution of the formation pitch drives the formation of a broadband reflective region of PSLC. Finally, the polymer network morphology of PD&SLC is demonstrated, and the adhesive strength of the films is verified.

## 2. Materials and Methods

### 2.1. Materials

**Figure 1:**
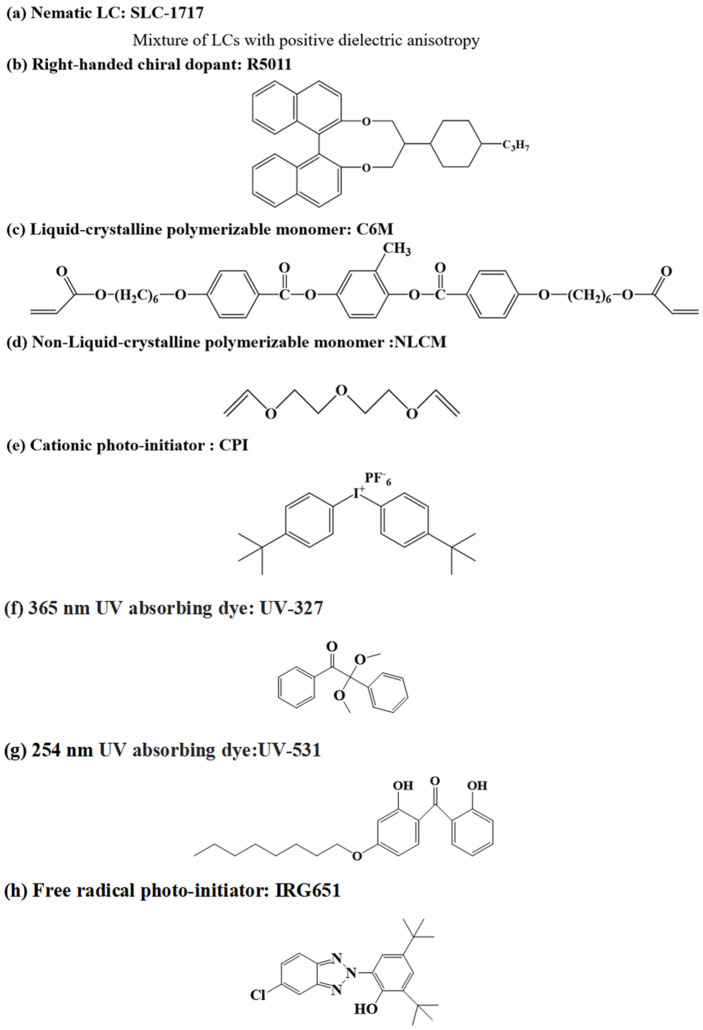
Chemical structure of the materials used.

The nematic liquid crystal SLC-1717 was provided by Shijiazhuang Chengzhi Yonghua Display Material Co., Ltd. (Shijiazhuang, China) (n_e_ = 1.720, n_o_ = 1.519, Δn = (n_e_ − n_o_) = 0.201), and the liquid crystal polymerizable monomer C6M was purchased from Beijing Kexin Jingyuan Electronics Co., Ltd. (Beijing, China). The non-liquid crystal polymerizable monomer NLCM was purchased from TCI Co., Ltd. (Taipei, Taiwan) The chiral compound R5011 was provided by Shijiazhuang Chengzhi Yonghua Display Material Co. Ltd. The 254 nm UV-absorbing dye UV-531 was provided by Shanghai Macklin Biochemical Co. Ltd. (Shanghai, China). The 365 nm UV-absorbing dye 2,4-Di-tert-butyl-6-(5-chloro-2H-benzotriazol-2-yl)phenol (UV-327) was purchased from Annaiji Chemical Reagent Co., Ltd. (Shanghai, China) The free radical photo-initiator 2,2-dimethopxy-1,2-diphenyl-ethanone (IRG651) was provided by TCI Co., Ltd. Cationic photo-initiators (CPI, TCI Co., Ltd.) were used in this study. The chemical structure of these materials is shown in Figure 1.

### 2.2. Preparation of Samples and Cells

Firstly, 3 g of polyvinyl alcohol (PVA) white solid was put into 97 mL of deionized water and slowly heated to 90 °C while stirring magnetically to obtain a solution of PVA at a concentration of 3% when there were all dissolved. The PET substrate was spin-coated with PVA solution by using the spin-coater at a maximum speed of 2000 r/min for 30 s, then thermally cured and placed in a hot oven at 50 °C for 2 h. The side was coated with the PVA orientation layer, and it was rubbed 3 times in a directional manner with a flannel, trying to avoid excessive force.

The nematic liquid crystal SLC-1717, liquid crystal polymerizable monomer C6M, non-liquid crystal polymerizable monomer NLCM, chiral compound R5011, 254 nm UV-absorbing dye UV-531, 365 nm UV-absorbing dye UV-327, cationic photo-initiator CPI, and free radical photo-initiator IRG651 were mixed according to A1-C6 in Table 1. Afterwards, a small amount of glass beads was added to control the thickness of the liquid crystal cell, and the uniformly mixed N*-LCs were obtained by heating dissolution and impact sonication.

**Figure 2:**
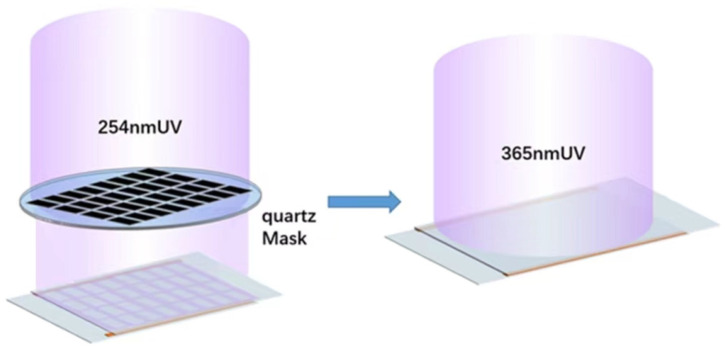
Schematic diagram of the step-by-step photocuring preparation of PD&SLC.

The blended liquid crystal system was poured between the two substrate layers and rolled into a thin film with a laminator. As shown in Figure 2, the lattice of quartz masks was placed on top of the film material and exposed to UV. The first step was a cationic UV curing technique, with UV curing at 254 nm to form PDLC, where the main purpose was to increase the bond strength of the film to the substrate material and the stability of the PSLC in the lattice. After the first step, the film was irradiated with 365 nm UV for the second step of free-radical UV curing, resulting in the formation of PD&SLC with reflective broadband.

### 2.3. Measurements

Bonding strength of PD&SLC to PET flexible substrate was measured by the electronic universal testing machine (SENS CMT4503). The optical textures of the samples were studied by using a POM (Olympus BX-51). The spectra of selective transmission were obtained using an UV/VIS/NIR-spectrophotometer (JASCO V-570). In general, Δλ is defined as the bandwidth at half the height of the transmitted light peak. The microscopic network morphology of PDLC and PD&SLC was observed by scanning electron microscope (SEM) (ZEISS SUPRA55).

## 3. Results and Discussion

### 3.1. Texture

**Figure 3:**
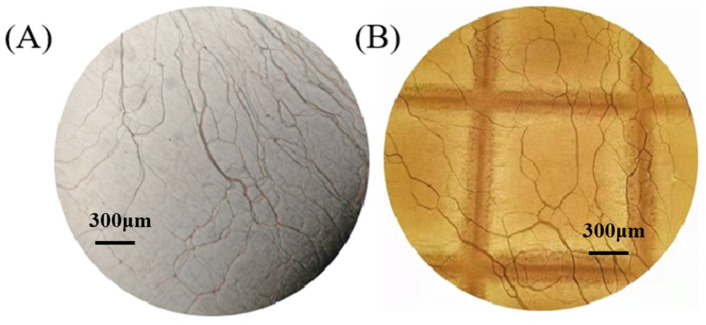
Texture composition before (**A**) and after curing (**B**).

In order not to destroy the conditions for selective reflection of N*-LC during UV curing, it is first necessary to ensure that N*-LC maintains a planar configuration after the first step of cationic light curing. Figure 3 shows the planar texture of N*-LC before light curing and after the completion of cationic and radical light curing, forming a local PD&SLC. A planar texture with a masking grid imprint is formed, which is not disrupted to form other textural states, such as the appearance of a focal cone texture, a fingerprint texture, or a hybrid texture. In a perfect planar texture, the helical axis of N*-LCs is perpendicular to the cell surface. Although the formation of planar textures does not guarantee the selective reflection property of N*-LCs, it is still an important step to ensure the selective reflection of N*-LCs. This also provides critical technical support for demonstrating that step-by-step photocuring via mask can be used to prepare flexible reflective broadband liquid crystal materials.

### 3.2. Mechanism of Broadband Reflection

**Figure 4:**
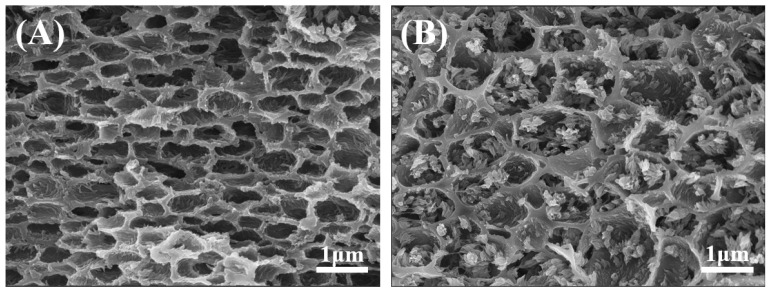
SEM pictures of PDLC (**A**) and PD&SLC (**B**).

In terms of raw materials, the major essential difference between PDLC and PSLC systems is the difference in the type of polymerizable monomers used. Non-liquid crystal polymerizable monomers are used in PDLC, and liquid crystal polymerizable monomers are used in PSLC. The lattice-like PDLC polymer wall not only increases the stabilization effect of PSLC liquid crystal molecules within the lattice and maintains the selective reflection effect of PSLC, but it also acts as a compression resistance within the film and increases the bonding strength of the film to the substrate. In order to obtain lattice-like PDLC polymer walls, simultaneous curing of both monomers must be avoided, so the step-by-step curing method is required. Cationic curing is performed with UV at 254 nm for 10 min in the first step, and free radical curing is performed with UV at 365 nm for 15 min at 40 °C in the second step. Therefore, the constructed PD&SLC combines the advantages of both PDLC and PSLC. Figure 4A shows the polymer network SEM picture of the honeycomb PDLC, from which it can be seen that the PDLC contains discontinuous cavities, which allow liquid crystal molecules to be dispersed in it. The liquid crystal polymer network of PSLC is an interwoven network rather than the circular hole morphology [30]. The SEM picture of the polymer network of PD&SLC in this study after cationic curing and free radical curing is shown in Figure 4B. The liquid crystal molecular system is dispersed in the pores of the PDLC, while the liquid crystal polymer network can help rivet the orientation of the liquid crystal molecules. It keeps the orientation of the helical axis of the N*-LCs in a perpendicular position to the substrate, that is, it can selectively reflect circularly polarized light.

**Figure 5:**
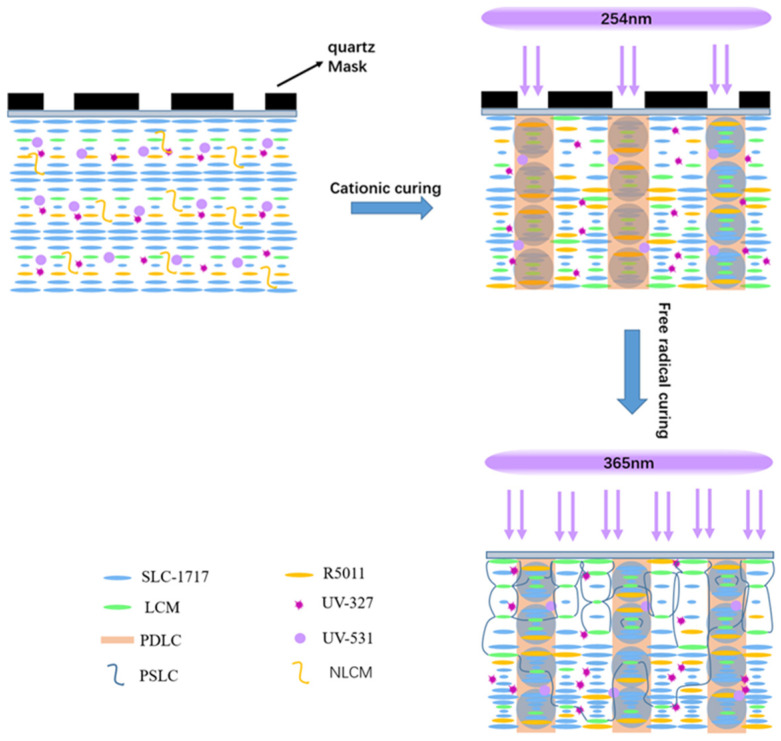
Schematic of the mechanism of broadband reflection.

As shown in Figure 5, the first step is the cationic curing of the 254 nm UV through the mask plate to form the PDLC walls. Flexibility can be achieved due to the high bonding strength of the PDLC to the substrate and the grid-like PDLC that effectively prevents the liquid crystal from flexing in the material. Meanwhile, due to the presence of 254 nm UV absorbing dye, the masked non-liquid crystal monomers, which are not irradiated by 254 nm UV, gradually migrate toward the irradiated region while further raising the density of PDLC. As the monomer migrates, the concentration gradient of the chiral compound R5011 is also formed in the UV intensity gradient so that the Δλ is broadened [31].

In the second step, free-radical curing with 365 nm UV light forms a PSLC region with broadband reflective properties. The region can be further broadened by adjusting the UV-absorbing dye content and irradiation conditions. The consumption of liquid crystal monomers is greater on the near-light side than on the far-light side, so that a large number of monomers migrate to the near-light side during UV light curing when the chiral compound R5011 migrates to the far-light side. A gradient distribution with a large pitch on the near-light side and a small pitch on the far-light side is formed, resulting in the Δλ being broadened once again.

### 3.3. Broadened Reflection Induced by the Content of UV-531

**Figure 6:**
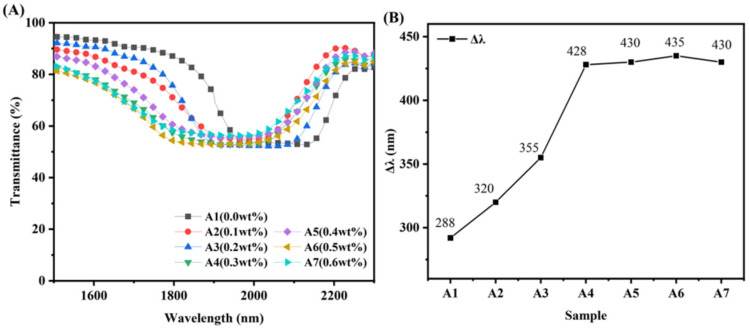
Transmission spectra (**A**) and Δλ of samples A1–A7 (**B**).

As shown in Figure 6, A1–A7 represent the variation of Δλ after the first cationic curing step for UV-531 concentrations of 0.0 wt%, 0.1 wt%, 0.2 wt%, 0.3 wt%, 0.4 wt%, 0.5 wt%, and 0.6 wt%, respectively. Curing and the relatively small migration of molecules in the small area of cation curing occur, forming a small but significant trend of Δλ broadening. A small difference in the intensity of the 254 nm UV light produced in the liquid crystal cassette thickness direction is observed at a smaller amount of UV-531. Thus, the polymerizable monomer is consumed relatively uniformly. A smaller amount of R5011 migration is generated in the liquid crystal cassette thickness direction, so the concentration difference of R5011 is smaller, and the amount of Δλ broadening is also smaller. As the content of UV-531 increased to 0.5 wt%, the difference in intensity of the 254 nm UV produced became increasingly large, so that the amount of Δλ being broadened gradually rose. However, with the continued increase of UV-531 content, the effect of Δλ being broadened tends to be constant. This is due to the relatively small UV area of 254 nm through the mask, and the overall consumption of non-liquid crystal polymerizable monomers is limited, and the amount of molecular migration is limited. Eventually, the amount of Δλ being widened cannot continue to increase.

### 3.4. Broadened Reflection Induced by the Content of C6M

**Figure 7:**
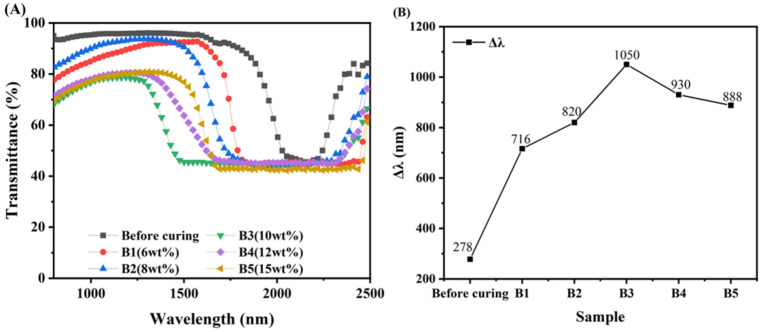
Transmission spectra (**A**) and Δλ of samples B1–B5 (**B**).

As shown in Figure 7, B1–B5 are the UV transmission spectra of the Δλ before the first step of cationic light curing and after the second step of free radical light curing when the content of C6M is explored at 6 wt%, 8 wt%, 10 wt%, 12 wt%, and 15 wt%, respectively. In Figure 7A, the effect of the Δλ being broadened by the second step of light curing can be clearly seen. When the content of C6M is 6 wt%, a gradient of UV intensity is created in the direction of the liquid crystal cartridge thickness due to the presence of UV-327 so that the near-light side is stronger than the far-light side. Uneven consumption of C6M in the direction of the cell thickness occurs during curing, and the amount of C6M consumed is greater on the near light side than on the far light side. C6M continuously migrates towards the near light side, while R5011 gradually migrates towards the far light side, which leads to a difference in the concentration of R5011 in the direction of the cell thickness, and eventually a gradient distribution of pitches is created. The content of R5011 on the near-light side is less than on the far-light side, and the liquid crystal pitch is larger on the near-light side and smaller on the far-light side. The Δλ of the samples increased with increasing C6M content, which is consistent with the results reported by Guillard and Sixou et al. [32,33].

When the content of C6M is low, the increase of C6M content favors the migration of C6M and the increase of curing rate, and the Δλ is gradually broadened [34]. However, when the C6M content exceeds a certain value, its excessively fast curing rate makes the C6M cured before it can migrate. This makes the pitch gradient of the system inevitably decrease, and Δλ gradually reduces. The reflective broadband reaches its maximum when the C6M content is 10 wt%. The rate of monomer curing is greater than the rate of migration at an increased C6M content of 12 wt%, allowing the monomer to be cured before migration is complete. A smaller amount of monomer migration results in a smaller pitch gradient formed by the liquid crystal molecules, which broadens Δλ by a smaller amount.

The Δλs of samples B1-B5 are shown in Figure 7B. The influence of the C6M content on the Δλ can be seen based on its trend. Other conditions being constant, the migration rate of the polymerizable monomer increases with increasing C6M content when the C6M content is less than 10 wt%. The rate at which polymerizable monomers are cured is greater than the rate of migration in the case of C6M content above a certain value, which is not conducive to the migration of monomers to form concentration differences. Thus, with regards to C6M at 10 wt%, the UV curing in the second step facilitates a broadening of the Δλ.

### 3.5. Broadened Reflection Induced by the Content of R5011

**Figure 8:**
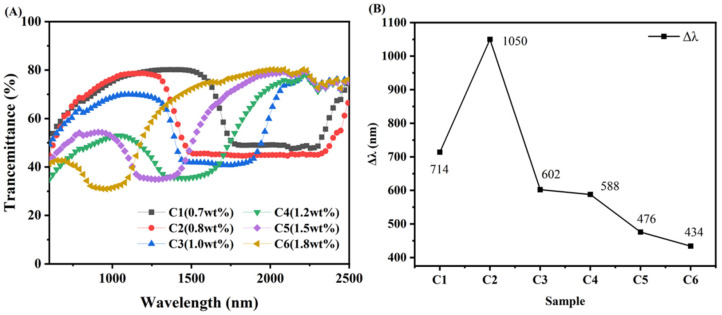
Transmission spectra (**A**) and Δλ of samples C1–C6 (**B**).

Figure 8 show the transmission spectra of samples C1–C6. Due to the higher consumption of polymerizable monomers on the near-light side, R5011 migrates towards the far-light side. R5011 is a chiral compound with a high helical distortion force, and the Δλ is shaped by minute variations in its content. The difference in concentration of R5011 formed in the direction of the cell thickness of the liquid crystal is small at the low content of R5011, so the Δλ is not broadened as effectively. This gradient difference in concentration gradually increases with increasing levels of R5011. This means that smaller pitch liquid crystal molecules are formed on the near light side, and larger pitch liquid crystal molecules are formed on the far light side. Therefore, a more obvious amount of Δλ is widened. However, the concentration gradient difference of R5011 becomes smaller instead regarding the content of R5011 above a certain value. As shown in Figure 8, the Δλ is 720 nm when the content of R5011 is 0.7 wt%. As the content of R5011 increases to 0.8 wt%, the Δλ reaches a maximum of 1050 nm and then starts to decrease gradually. This result is consistent with that reported by Guillard and Sixou et al. [24,25]. In addition, a significant blue shift in λm occurs with increasing R5011 content, meaning that the λm of the selective reflection can be modulated by controlling the R5011 content.

### 3.6. Broadened Reflection Induced by the UV Intensity

**Figure 9:**
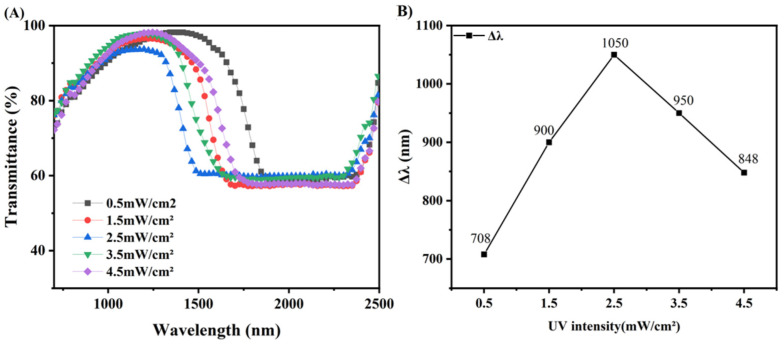
Transmission spectra (**A**) and Δλ for different UV intensities (**B**).

Figure 9 shows the sample transmission spectra obtained by changing different UV light intensities under the condition that other conditions are exactly the same as B3. As shown in Figure 9, as the UV intensity (365 nm) increases from 0.5 mW/cm^2^ to 4.5 mW/cm^2^, Δλ first increases from 708 nm to 1050 nm. However, as the UV intensity continues to increase, the Δλ drops back down to 848 nm. Since UV-absorbing dyes work by absorbing UV-intensity, increasing the content of UV-absorbing dyes and increasing the intensity of UV can, in a sense, have the same effect.

Initially, the gradient of UV intensity increases with increasing UV intensity, with the migration of C6M molecules playing a dominant role, so that the Δλ changes with increasing UV intensity, a result consistent with that reported by Guillard and Sixou et al. [24,25]. However, as the UV intensity increases to a certain value, the gradient of the UV intensity decreases with increasing UV intensity. The curing of C6M is accelerated, and the polymer network formed after curing hinders the migration of molecules, and the tendency of the sample to broaden the Δλ is reduced.

### 3.7. Comparison of Bonding Strength

**Figure 10:**
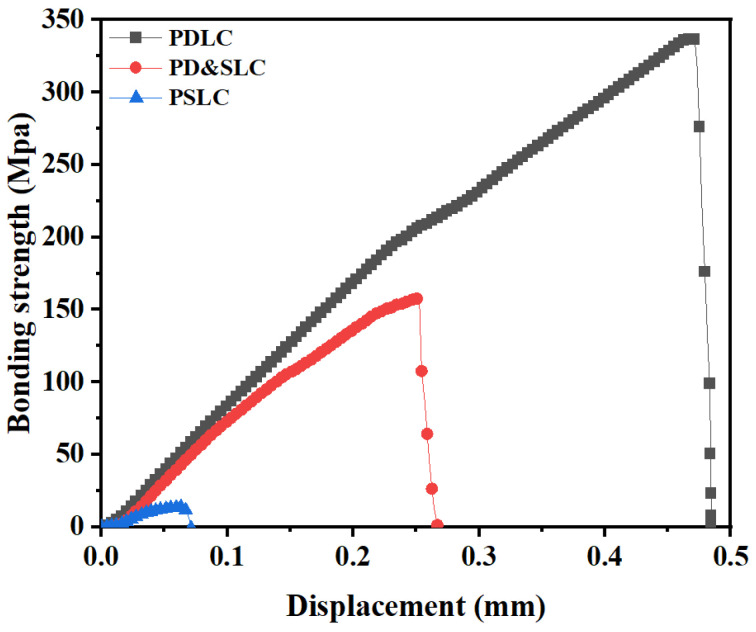
Bonding strength of PDLC, PD&SLC and PSLC.

After the first step of cationic UV curing and the second step of free radical UV curing, PD&SLC is formed. In this study, the cross-sectional network morphology of the three films, PDLC, PD&SLC, and PSLC, was examined. The bond strength of the PET flexible substrate was also further tested using an electronic universal testing machine, as shown in Figure 10. The parameter size of the specimen was: 20 mm × 25 mm × 0.04 mm, which was entered into the controlled computer; the speed of the test displacement was adjusted to 4.0 mm/min, and the bond strength formula is P = F/S (where P represents the bond strength, F represents the shear stress, and S represents the area of the film subjected to the force). The PDLC contains 40 wt% of polymerizable monomers, the PD&SLC contains 26 wt% of polymerizable monomers, and the PSLC contains 10 wt% of polymerizable monomers. PDLC not only contains a high content of polymerizable molecules in the non-liquid crystal polymerizable monomer, but also forms a denser polymer network with stronger bonding strength to the substrate. With a four-fold difference in polymerizable monomer content, PDLC has more than 15 times higher bond strength than PSLC. This indicates that NLCM forms a stronger polymer network than C6M. PD&SLC, on the other hand, exhibits intermediate bond strengths, but this is still nearly nine times higher than PSLC for the preparation of flexible film properties.

## 4. Conclusions

This paper presents a novel and effective method to develop a flexible and reflective broadband liquid crystal film based on the PD&SLC. Based on the coexistence of these two polymer networks, the new system has a lattice-like PDLC polymer wall. This effectively prevents the mobility of the liquid crystals from flexing in the material, increasing the stabilizing effect of the PSLC liquid crystal molecules. The selective reflective effect of PSLC is retained, while broadening of the reflective broadband is achieved. By systematically studying the effects of the content of UV absorbing dyes, polymerizable monomers, chiral monomers, and UV intensity on the Δλ of PD&SLC, a broadening of the Δλ up to 1050 nm in the near infrared region was achieved. The advantage of PD&SLC in flexible film performance was also verified by a bonding strength test. In summary, flexible and reflective broadband liquid crystal films can be prepared by cation–radical stepwise photocuring. It is believed that this study will be of great significance to the development of cholesteric liquid crystal composites.

## Figures and Tables

**Table 1:** Compositions of the samples studied.

Sample Number	SLC-1717 (wt%)	C6M (wt%)	NLCM (wt%)	UV-327 (wt%)	UV-531 (wt%)	IRG651 (wt%)	CPI (wt%)	R5011 (wt%)
A1	72.5	10	16	0.3	0	0.2	0.2	0.8
A2	72.4	10	16	0.3	0.1	0.2	0.2	0.8
A3	72.3	10	16	0.3	0.2	0.2	0.2	0.8
A4	72.2	10	16	0.3	0.3	0.2	0.2	0.8
A5	72.1	10	16	0.3	0.4	0.2	0.2	0.8
A6	72	10	16	0.3	0.5	0.2	0.2	0.8
A7	71.9	10	16	0.3	0.6	0.2	0.2	0.8
B1	76.2	6	16	0.3	0.3	0.2	0.2	0.8
B2	74.2	8	16	0.3	0.3	0.2	0.2	0.8
B3	72.2	10	16	0.3	0.3	0.2	0.2	0.8
B4	70.2	12	16	0.3	0.3	0.2	0.2	0.8
B5	67.2	15	16	0.3	0.3	0.2	0.2	0.8
C1	72.3	10	16	0.3	0.3	0.2	0.2	0.7
C2	72.2	10	16	0.3	0.3	0.2	0.2	0.8
C3	72	10	16	0.3	0.3	0.2	0.2	1
C4	71.8	10	16	0.3	0.3	0.2	0.2	1.2
C5	71.5	10	16	0.3	0.3	0.2	0.2	1.5
C6	71.2	10	16	0.3	0.3	0.2	0.2	1.8

## Data Availability

Data are contained within the article.

## References

[1] Drzaic P. (1986). Polymer dispersed nematic liquid crystal for large area displays and light valves. J. Appl. Phys..

[2] Dierking I., Scalia G., Morales P., LeClere D. (2004). Aligning and Reorienting Carbon Nanotubes with Nematic Liquid Crystals. Adv. Mater..

[3] Khandelwal H., Schenning A.H.J., Debije M.G. (2017). Infrared Regulating Smart Window Based on Organic Materials. Adv. Energy Mater..

[4] Dierking I., Scalia G. (2005). Liquid crystal–carbon nanotube dispersions. J. Appl. Phys..

[5] Kim D.J., Hwang D.Y., Park J.Y., Kim H.K. (2018). Liquid crystal–Based flexible smart windows on roll-to-roll slot die—Coated Ag nanowire network films. J. Alloys Compd..

[6] Hinojosa A., Sharma S.C. (2010). Effects of gold nanoparticles on electro-optical properties of a polymer-dispersed liquid crystal. Appl. Phys. Lett..

[7] Dierking I. (2000). Polymer Network–Stabilized Liquid Crystals. Adv. Mater..

[8] Mitov M., Nouvet E., Dessaud N. (2004). Polymer-stabilized cholesteric liquid crystals as switchable photonic broad bandgaps. Eur. Phys. J. E.

[9] Lee M.H., Li X.D., Kim Y.J., Kang J., Paek S., Kim J.J. (2002). Facile Fabrication of Polymer Waveguide by Using Photosensitive Polyimides. Mol. Cryst. Liq. Cryst..

[10] Chen X.W., Wang L., Chen Y.J., Li C.Y., Hou G.Y., Liu X., Zhang X.G., He W.L., Yang H. (2014). Broadband reflection of polymer-stabilized chiral nematic liquid crystals induced by a chiral azobenzene compound. Chem. Commun..

[11] St. John W.D., Fritz W.J., Lu Z.J., Yang D.K. (1995). Bragg reflection from cholesteric liquid crystals. Phys. Rev. E.

[12] Boudet A., Binet C., Mitov M., Bourgerette C., Boucher E. (2000). Microstructure of variable pitch cholesteric films and its relationship with the optical properties. Eur. Phys. J. E.

[13] Guo J.B., Sun J., Zhang L.P., Li K.X., Cao H., Yang H., Zhu S.Q. (2008). Broadband reflection in polymer stabilized cholesteric liquid crystal cells with chiral monomers derived from cholesterol. Polym. Adv. Technol..

[14] Broer D.J., Lub J., Mol G.N. (1997). Photo-controlled diffusion in reacting liquid crystals: A new tool for the creation of complex molecular architectures. Macromol. Symp..

[15] Broer D.J., Mol G.N., Haaren J.M.M., Lub J. (1999). Photo-induced diffusion in polymerizing chiral-nematic media. Adv. Mater..

[16] Hu W., Chen M., Wang Q., Zhang L.Y., Yuan X.T., Chen F.W., Yang H. (2019). Broadband reflection in polymer-stabilized cholesteric liquid crystals via thiol–acrylate chemistry. Angew. Chem. Int. Ed..

[17] Zhao Y.Z., Zhang L.Y., He Z.M., Chen G., Wang D., Zhang H.Q., Yang H. (2015). Photoinduced polymer-stabilised chiral nematic liquid crystal films reflecting both right- and left-circularly polarised light. Liq. Cryst..

[18] Guo J.B., Cao H., Wei J., Zhang D.W., Liu F., Pan G.H., Zhao D.Y., He W.L., Yang H. (2008). Polymer stabilized liquid crystal films reflecting both right- and left-circularly polarized light. Appl. Phys. Lett..

[19] Guo J., Yang H., Li R., Ji N., Dong X.M., Wu H., Wei J. (2009). Effect of Network concentration on the performance of polymer-stabilized cholesteric liquid crystals with a double-handed circularly polarized light reflection band. J. Phys. Chem. C.

[20] Guo J., Liu F., Chen F.J., Wei J., Yang H. (2010). Realisation of cholesteric liquid-crystalline materials reflecting both right- and left-circularly polarised light using the wash-out/refill technique. Liq. Cryst..

[21] Li Y., Liu Y.J., Luo D. (2017). Optical thermal sensor based on cholesteric film refilled with mixture of toluene and ethanol. Opt. Express.

[22] Zhang L.S., Pan J.K., Liu Y.H., Xu Y., Zhang A.M. (2020). NIR–UV responsive actuator with graphene oxide/microchannel-induced liquid crystal bilayer structure for biomimetic devices. ACS Appl. Mater. Interfaces.

[23] Hwang H., Kang M.S., Han J.H., Shin K., Cho J.H. (2013). Photo-crosslinkable NIR-absorbing window with environmental stability. Pigm. Resin. Technol..

[24] Kim H., Lee J.A., Ambulo C.P., Lee H.B., Kim S.H., Naik V.V., Haines C.S., Aliev A.E., Ovalle-Robles R., Baughman R.H. (2019). Intelligently actuating liquid crystal elastomer-carbon nanotube composites. Adv. Funct. Mater..

[25] Bakker L.G., Brouwer A.H., Babuska R. (2001). Integrated smart control of heating, cooling, ventilation, day-lighting and electrical lighting in buildings. J. Sol. Energy.

[26] Kolvir H.R., Domola H.M. (2015). The study of environmental psychology in tall buildings with sustainable architecture approach. Toxicol. Appl. Pharm..

[27] Liang X., Chen M., Wang Q., Guo S.J., Yang H. (2019). Ethanol-Precipitable, Silica-Passivated perovskite nanocrystals incorporated into polystyrene microspheres for long-term storage and reusage. Angew. Chem. Int. Ed..

[28] Liang X., Chen M., Wang Q., Guo S.M., Zhang L.Y., Yang H. (2018). Active and passive modulation of solar light transmittance in a hybrid thermochromic soft-matter system for energy-saving smart window applications. J. Mater. Chem. C.

[29] Guo S.M., Liang X., Zhang C.H., Chen M., Shen C., Zhang L.Y., Yang X., He B.F., Yang H. (2017). Preparation of a thermally light-transmittance-controllable film from a coexistent system of polymer-dispersed and polymer-stabilized liquid crystals. ACS Appl. Mater. Interfaces.

[30] Wang F.F., Li K.X., Song P., Wu X.J., Cao H., Yang H. (2012). Photoinduced pitch gradients and the reflection behaviour of the broadband films: Influence of dye concentration, light intensity, temperature and monomer concentration. Liq. Cryst..

[31] Zhang X.T., Shi W.T., Han R., Li H., Cao H., Chen Y.J., Yang Z., Wang D., He W.L. (2022). Self-diffusion method for broadband reflection in polymer-stabilized cholesteric liquid crystal films. Liq. Cryst..

[32] Guillard H., Sixou P. (2001). Active broadband polymer stabilized liquid crystals. Liq. Cryst..

[33] Guillard H., Sixou P., Reboul L., Perichaud A. (2001). Electrooptical characterizations of polymer stabilized cholesteric liquid crystals. Polymer.

[34] Shi W.T., Zhang X.T., Han R., Li H., Cao H., Chen Y.J., Wang D., Yang Z., He W.L. (2022). Preparation of cholesteric polymer networks with broadband reflection memory effect. Liq. Cryst..

